# Correlation of Tc-99 m ethyl cysteinate dimer single-photon emission computed tomography and clinical presentations in patients with low cobalamin status

**DOI:** 10.1186/s12883-015-0500-4

**Published:** 2015-12-03

**Authors:** Min-Chien Tu, Chung-Ping Lo, Ching-Yuan Chen, Ching-Feng Huang

**Affiliations:** Department of Neurology, Taichung Tzu Chi Hospital, Buddhist Tzu Chi Medical Foundation, Taichung, Taiwan; Department of Radiology, Taichung Tzu Chi Hospital, Buddhist Tzu Chi Foundation, Taichung, Taiwan; Department of Nuclear Medicine, Taichung Tzu Chi Hospital, Buddhist Tzu Chi Medical Foundation, Taichung, Taiwan; School of Medicine, Tzu Chi University, Hualien, Taiwan; Graduate Institute of Medical Imaging and Radiological Sciences, Central Taiwan, University of Science and Technology, Taichung, Taiwan

**Keywords:** Cobalamin deficiency, Vitamin B12, Tc-99 m-ECD, SPECT, Cognition, Mild cognitive impairment

## Abstract

**Background:**

Cobalamin (Cbl) deficiency has been associated with various neuropsychiatric symptoms of different severities. While some studies dedicated in structural neuroimaging credibly address negative impact of low Cbl status, functional imaging reports are limited. We herein retrospectively review the correlation of Tc-99 m ethyl cysteinate dimer single-photon emission computed tomography (Tc-99 m-ECD SPECT) and clinical presentations among patients with low serum cobalamin (Cbl) status (<250 pg/ml).

**Methods:**

Twelve symptomatic patients with low serum Cbl status were enrolled. Clinical presentations, Tc-99 m-ECD SPECT, and neuropsychological tests were reviewed.

**Results:**

Dysexecutive syndrome (67 %), forgetfulness (50 %), attention deficits (42 %), and sleep disorders (33 %) constituted the major clinical presentations. All patients (100 %) had temporal hypoperfusion on the Tc-99 m-ECD SPECT. Five patients (42 %) had hypoperfusion restricted within temporal regions and deep nuclei; seven patients (58 %) had additional frontal hypoperfusion. In patients with hypoperfusion restricted within temporal regions and deep nuclei, psychiatric symptoms with spared cognition were their main presentations. Among patients with additional frontal hypoperfusion, six of seven patients (86 %) showed impaired cognitive performances (two of them were diagnosed as having dementia). Among ten patients who finished neuropsychological tests, abstract thinking (70 %) was the most commonly affected, followed by verbal fluency (60 %), short-term memory (50 %), and attention (50 %). Anxiety and sleep problems were the major clinically remarkable psychiatric features (33 % both). Four Tc-99 m-ECD SPECT follow-up studies were available; the degree and extent of signal reversal correlated with cognitive changes after Cbl replacement therapy.

**Conclusions:**

Our TC-99 m-ECD SPECT observations provide pivotal information of neurobiological changes within basal ganglia and fronto-temporal regions in conjunction with disease severity among patients with Cbl deficiency. Hypoperfusion within thalamus/basal ganglia and temporal regions may be seen in the earlier state of Cbl deficiency, when psychiatric symptoms predominate. Hypoperfusion beyond thalamus/basal ganglia and involving frontal regions appears when cognitive problems, mostly dysexecutive syndrome, are manifested. Symmetric hypofrontality of SPECT in the context of dysexcutive syndrome serves as a distinguishing feature of non-amnestic mild cognitive impairment attributed to Cbl deficiency. Concordant with TC-99 m-ECD SPECT findings, the psychiatric symptoms and dysexcutive syndrome undergird impaired limbic and dorsolateral prefrontal circuits originating from basal ganglia respectively.

## Background

Cobalamin (Cbl) deficiency has been associated with various neuropsychiatric symptoms of different severities [1]. Delayed detection of Cbl deficiency among the elderly is sometimes complicated with severe neurological consequences; hence determination of Cbl status is often included in the diagnostic workup for dementia [2]. Serology evaluations of Cbl level are also frequently enlisted as part of diagnostic repertoire for mild cognitive impairment (MCI), an increasingly-identified syndrome in which changes of cognition exist but not to the extent of dementia [3]. Although previous literatures have addressed the negative impact of low Cbl status on myelin formation, cellular membrane stability, and neurotransmitters regulation [4], the causality between cognition decline and Cbl status remains inconclusive [1]. As subtle neuropathological changes in the brain may precede profound symptoms or clinical dementia, neuroimaging study provides a useful tool to probe fundamental mechanisms of disease process. While some studies dedicated in structural neuroimaging credibly address negative impact of low Cbl status [5, 6], functional imaging reports are limited [7, 8]. Therefore, we conducted a surveillance to integrate the clinical presentations and Tc-99 m ethyl cysteinate dimer single-photon emission computed tomography (Tc-99 m-ECD SPECT) among patients with low Cbl status.

## Methods

We retrospectively reviewed twelve symptomatic patients with low serum Cbl status (<250 pg/ml) [9]. Their main symptoms were featured with cognitive (e.g., forgetfulness and difficulty on planning) and/or psychiatric (e.g., sleep problems and low mood) complaints. All patients had checked complete blood count, thyroid function, cortisol, serum folic acid levels, and rapid plasma reagin screen, which were within normal limits. All patients received brain magnetic resonance imaging (MRI) for excluding cerebral vascular occlusion and/or large cortical infarcts, which would be possible confounding factors to SPECT observations. Tc-99 m-ECD SPECT scan interpretations and clinical profiles, including demographic data and neuropsychological tests, were collected. The written informed consent was obtained from all participants and there relevant radiation exposure was under regulation of radiometry in our country. The study was permitted by the Institutional Review Board of the Taichung Tzu Chi Hospital, Buddhist Tzu Chi Medical Foundation (REC 103–47).

### Tc-99 m-ECD SPECT

Twelve patients underwent a Tc-99 m-ECD SPECT scan at rest, conducted before or one week within the start of Cbl supplement. A brain scintigraphy was performed 30 min after intraveneous injection of 740 Mbq 99mTc- ECD on General Electric, Infinia Hawkeye 4. Subsequently, a combined SPECT equipped with a dual-head gamma-camera with low-energy high resolution collimators, a 7 % energy window centered at 140 keV and 128 × 128 matrix (GE, USA) was conducted. Acquisition was performed in continuous mode with 240 projections in a 128 × 128 matrix, zoom:1. The camera operated in segmented mode with double 360*2゜rotation with 3゜per projection angle. No scatter windows were placed. Through the mathematical approximation technique, acquired data were reconstructed (ordered-subset expectation maximization attenuation scatter method; Chang order 0; threshold 10; coefficient 0.11; cutoff frequency, 0.3 cycles/pixel for the main) after applying a Butterworth filter. The SPECT was displayed on GE Xeleris 2 workstation in axial, sagittal, and coronal slices. A count difference cost function with an iterative downhill-simplex search algorithm was applied for registration. The images were displayed on a computer monitor using an identical cool color scale (window, 100; base, 0).

**Fig. 1:**
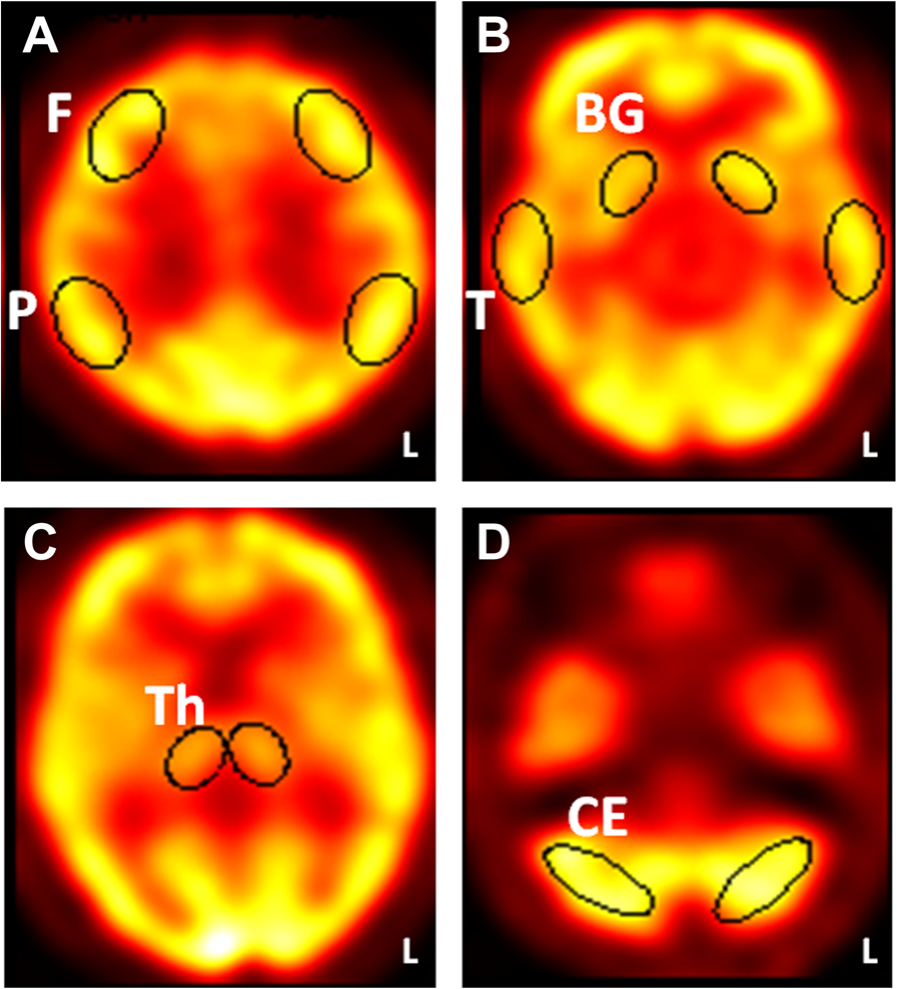
Regions-of-interest templates placed on selected axial images of single-photon emission computed tomography (SPECT). (**a**) F = frontal regions; P = parietal regions. (**b**) T = temporal regions; BG = basal ganglia. (**c**) Th = thalamus. (**d**) CE = cerebellum

Both visual and quantitative image analyses were performed. For visual analysis, images were reported independently by two nuclear medicine physicians who were blind to radiological and clinical results. Should there be any discrepancy between the interpretations, a consensus would be reached after panel discussion. For the quantitative analysis, 4 fixed axial images with predefined templates were selected (Fig. 1). Ten regions of interest, including six in bilateral cerebral cortices (frontal, temporal, and parietal regions) and four in deep nuclei (thalamus and basal ganglia), were symmetrically analyzed in each hemisphere. The regions of interest ranged in pixels from 90 (deep nuclei) to 170 (cerebral cortices) and were fixed in size across studies. All regions of interest were placed by the same operator to eliminate interoperator variability. The intraoperator reliability in the quantitative measurements was found to be Kappa = 0.821 (*p* < 0.001), 95 % CI (0.602, 1.040) (*n* = 12 times). The mean counts in each selected region were normalized with respect to the mean counts in the ipsilateral cerebellum to quantify regional ECD uptake. In order to represent regional signal changes and avoid selection bias, final ECD uptake would be calculated by averaging data in which templates were placed onto three sequential neighboring slices. The average values of regions of interest to cerebellum ratio less than 0.8 were defined as abnormal and recorded [10].

### Neuropsychological tests

Neuropsychological tests were available in ten patients. Mini-mental state examination (MMSE) [11], Cognitive Abilities Screening Instrument (CASI) [12], Clinical Dementia Rating (CDR) [13], and neuropsychiatric inventory (NPI) [14] were thoroughly reviewed. In both MMSE and CASI, higher score represents better cognitive achievement. A MMSE score less than 26 would conventionally be classified as indicative of cognitive impairment [11]. CASI total scores below the cut-off score of normative data in Taiwan were registered as abnormal [15]. Additionally, CASI provides quantitative assessment of 9 cognitive subdomains, including long term memory, short term memory, attention, mental manipulation, orientation, abstract thinking, language abilities, drawing, and verbal fluency. Scores of CASI subdomain falling more than one standard deviation of normative data in Taiwan were registered as abnormal [15]. CDR was applied to evaluate the daily functional performance related with the severity of cognitive deficits (0 = Normal; 0.5 = Very mild; 1 = Mild; 2 = Moderate; 3 = Severe). NPI, in which twelve psychological symptoms were determined, were registered clinically-remarkable if score of each defined symptom more than 4 [14].

## Results

### Tc-99 m-ECD SPECT

Tc-99 m-ECD SPECT findings in line with clinical profiles were summarized in Table 1. The patients’ age ranged from 29 to 81. Dysexecutive syndrome (67 %), forgetfulness (50 %), attention deficits (42 %), and sleep disorders (33 %) constituted the major clinical presentations. All patients (100 %) had temporal hypoperfursion on the Tc-99 m-ECD SPECT. Five patients (42 %) (Patient #8 ~ #12) had hypoperfusion restricted within temporal regions and deep nuclei; seven patients (58 %) (Patient #1 ~ #7) had frontal hypoperfusion in addition to temporal hypoperfusion. In patients with hypoperfusion restricted within temporal regions and deep nuclei, their cognitive performances were intact (MMSE = 26 ~ 27; CASI = 75 ~ 93) and daily functional performance were relatively preserved (CDR = 0 ~ 0.5). Three of five patients (60 %) presented with psychiatric symptoms (i.e., irritability, anxiety, and sleep disorders). Among patients with additional frontal hypoperfusion, six of seven patients (86 %) showed impaired cognitive performances (MMSE = 12 ~ 25; CASI = 34 ~ 82) and impaired daily functional performance (CDR = 0.5 ~ 1). Only one patient had normal achievement of neuropsychological tests and intact daily functional status (Patient #7). Dysexecutive syndrome (86 %) and attention deficits (57 %) constituted the major clinical presentations. Anxiety and insomnia were recorded in three patients (43 %). Two patients (29 %) were eventually diagnosed with dementia.

**Table 1:** Demographic data and single-photon emission computed tomography observation of patients with cobalamin deficiency

Patient #	Age/Gender	Education level (years)	Serum cobalamin level (pg/ml)	Clinical Dementia Rating	Mini-Mental State Examination	Cognitive Abilities Screening Instrument	Single-photon emission computed tomography	Clinical presentations
1	73/F	0	199	1	12	34	Bil. F. T. P. Tha. (more severe in Lt) Lt BG.	Forgetfulness, attention deficits, dysexcutive syndrome, and dementia
2	80/F	0	230	1	13	42	Bil. F. T. Tha. Rt BG	Forgetfulness and dementia
3	60/F	6	207	0.5	24	78	Bil. F. T. Tha.	Insomnia, forgetfulness, attention deficits, and dysexcutive syndrome
4	81/M	12	177	0.5	25	82	Bil. F. T. Tha.BG (more severe in Rt)	Depression, forgetfulness, attention deficits, and dysexcutive syndrome
5	53/F	6	218	0.5	22	65	Bil. F. T. Tha.	Dysexecutive syndrome, anxiety, and insomnia
6	74/F	6	209	0.5	21	61	Bil. F. T. Tha.	Forgetfulness, attention deficits, dysexcutive syndrome, and anxiety
7	58/F	12	210	0	28	88	Bil. F. T. Tha.	Anxiety, insomnia, and dysexcutive syndrome
8	56/F	12	229	0.5	27	87	Bil. T. Tha.	Dysexecutive syndrome
9	65/F	6	202	0.5	26	93	Bil. T. Rt Tha.	Attention deficits
10	61/M	6	108	0.5	27	75	Bil. T. (more severe in Lt) Lt BG	Irritability, forgetfulness, and dysexcutive syndrome
11	29/F	12	199	0	N.A.	N.A.	Lt T. Tha.	Irritability, anxiety, dysthymia, and sleep disorders
12	41/M	12	175	0	N.A.	N.A	Bil. T. Rt BG	Anxiety

Abbreviations: *F* Famale, *M*: Male, *N.A* non-available, *Bil* bilateral, *Rt* right, *Lt* left, *F* frontal region, *T* temporal region, *P* parietal region, *Tha* thalamus, *BG* basal ganglia

Quantitative assessment of Tc-99 m-ECD SPECT was detailed in Table 2. Data were presented as individual value of three consecutive slices and their average. From the data of cerebral cortices, abnormal ratio counts within temporal regions were identified in all patients (eleven as bilateral and one as unilateral involvement). Abnormal ratio counts within frontal regions were identified in seven patients (Patient #1-#7); all presented as bilateral involvement. From the data of deep nuclei, ten had impaired ratio count within thalamus (eight as bilateral and two as unilateral involvement). Five had impaired ratio count within basal ganglia.

**Table 2:** Regions-to-cerebellar ratio among patients with cobalamin deficiency

	Frontal regions						Temporal regions						Parietal regions						Thalamus						Basal ganglia					
	Rt			Lt			Rt			Lt			Rt			Lt			Rt			Lt			Rt			Lt		
Patient #																														
1	.81	.77	.78	.82	.80	.76	.80	.78	.79	.75	.75	.71	.79	.78	.79	.79	.79	.80	.76	.75	.79	.72	.73	.68	.82	.82	.81	.74	.74	.74
	**.79**			**.79**			**.79**			**.74**			**.79**			**.79**			**.77**			**.71**			**.82**			**.74**		
2	.78	.80	.80	.74	.74	.75	.79	.81	.78	.73	.74	.76	.95	.92	.95	.82	.82	.80	.72	.72	.71	.70	.71	.72	.72	.70	.71	.80	.81	.81
	**.79**			**.74**			**.79**			**.74**			**.94**			**.81**			**.72**			**.71**			**.71**			**.81**		
3	.76	.80	.81	.80	.80	.76	.75	.75	.76	.76	.78	.81	.99	1.01	.99	.95	.95	.99	.73	.72	.73	.68	.68	.68	.96	.95	.96	.92	.93	.93
	**.79**			**.79**			**.75**			**.78**			**1.00**			**.96**			**.73**			**.68**			**.96**			**.93**		
4	.68	.67	.72	.78	.77	.77	.76	.75	.76	.77	.78	.78	.89	.87	.85	.92	.95	.94	.73	.76	.76	.78	.78	.78	.61	.63	.67	.78	.77	.74
	**.72**			**.77**			**.76**			**.78**			**.87**			**.94**			**.75**			**.78**			**.64**			**.76**		
5	.76	.76	.80	.73	.80	.77	.75	.75	.71	.77	.76	.76	.82	.82	.86	.83	.82	.83	.77	.78	.78	.67	.66	.72	.83	.84	.83	.82	.82	.82
	**.77**			**.77**			**.74**			**.76**			**.83**			**.83**			**.78**			**.68**			**.83**			**.82**		
6	.75	.77	.80	.80	.75	.83	.78	.78	.80	.77	.76	.77	.95	.93	.94	.94	.95	.96	.78	.78	.80	.78	.79	.79	.89	.87	.87	.86	.85	.88
	**.77**			**.79**			**.79**			**.77**			**.94**			**.95**			**.79**			**.79**			**.88**			**.86**		
7	.78	.80	.76	.79	.77	.77	.75	.75	.74	.75	.75	.73	.85	.87	.85	.84	.81	.83	.67	.69	.69	.69	.69	.70	.91	.92	.94	.93	.95	.93
	**.78**			**.78**			**.75**			**.74**			**.86**			**.83**			**.68**			**.69**			**.92**			**.94**		
8	.84	.88	.87	.90	.92	.88	.75	.70	.70	.79	.79	.79	.90	.89	.89	.92	.92	.93	.71	.69	.72	.79	.82	.75	.91	.90	.87	.96	.95	.96
	**.86**			**.90**			**.72**			**.79**			**.89**			**.92**			**.71**			**.79**			**.89**			**.96**		
9	.86	.87	.85	.97	.92	.94	.77	.77	.77	.80	.79	.79	.82	.83	.82	.97	.94	.97	.66	.65	.64	.83	.83	.83	.94	.92	.92	.96	.99	.95
	**.86**			**.94**			**.77**			**.79**			**.82**			**.96**			**.65**			**.83**			**.93**			**.97**		
10	.90	.89	.88	.83	.83	.83	.79	.75	.76	.68	.74	.78	.98	.95	.98	.79	.86	.89	.80	.80	.80	.81	.81	.79	.85	.81	.88	.80	.79	.79
	**.89**			**.83**			**.77**			**.73**			**.97**			**.85**			**.80**			**.80**			**.85**			**.79**		
11	.93	.92	.86	.83	.88	.90	.82	.82	.81	.72	.72	.72	.98	1.02	1.00	.95	.85	.91	.86	.84	.80	.77	.72	.74	.98	.99	1.00	.95	.93	.95
	**.90**			**.87**			**.82**			**.72**			**1.00**			**.90**			**.83**			**.74**			**.99**			**.94**		
12	.86	.92	.94	.91	.90	.87	.78	.74	.76	.77	.71	.74	.98	.98	.98	.97	.97	.98	.81	.81	.81	.79	.80	.80	.75	.75	.75	.87	.84	.87
	**.91**			**.89**			**.76**			**.74**			**.98**			**.97**			**.81**			**.80**			**.75**			**.86**		

Abbreviations: *Rt* right, *Lt* left; data were presented as ratio count from three consecutive slices (serif) followed by their average value (boldface)

**Fig. 2:**
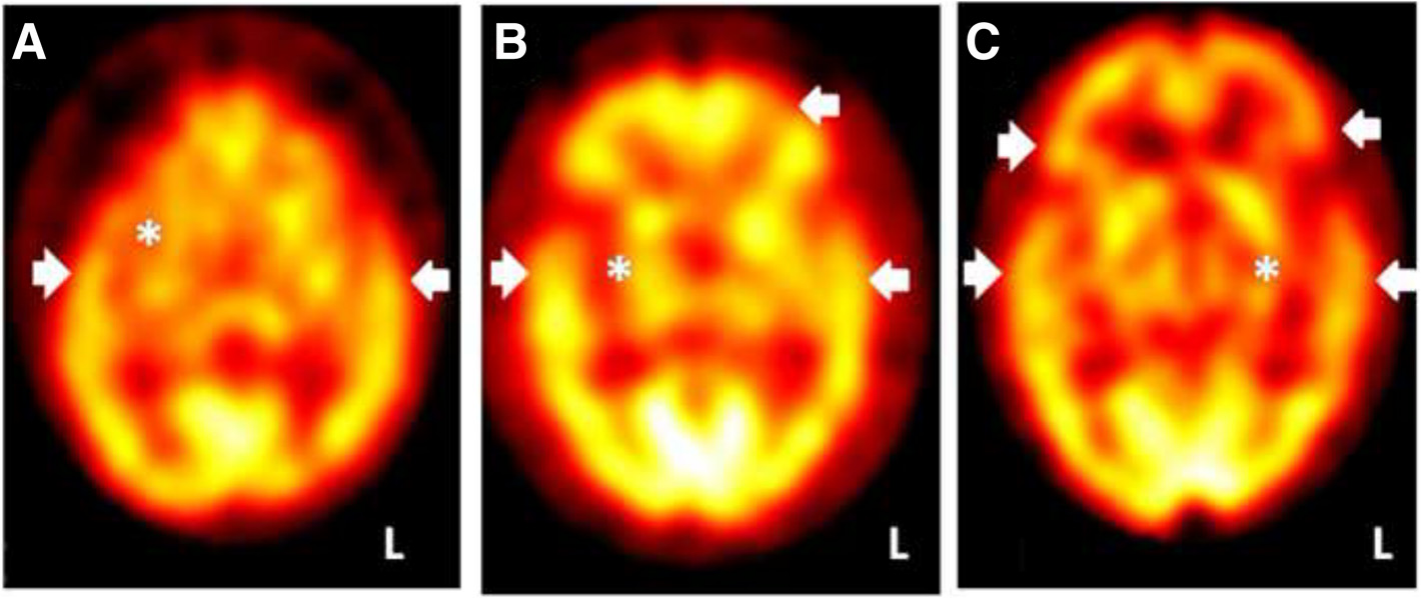
Single-photon emission computed tomography (SPECT) correlates with clinical presentations and disease severity of cobalamin-deficient patients. (**a**) A case with anxiety disorder but preserved cognition. Clinical Dementia Rating (CDR) = 0. SPECT shows hypoperfusion in right basal ganglia (asterisk) and bilateral temporal tips (arrow heads; Patient #12). (**b**) A case with prominent dysexecutive syndrome. CDR = 0.5. SPECT shows hypoperfusion in right basal ganglia (asterisk) and left dorsolateral prefrontal cortex, in addition to temporal regions. (arrow heads; Patient #4). (**c**) A case with clinical diagnosis of dementia. CDR = 1. SPECT shows bilateral, more pronounced in the left, thalamus (asterisk) and fronto-temporal regions hypoperfusion (arrow heads; Patient #1)

Intriguingly, Tc-99 m-ECD SPECT changed in accordance with different clinical severity. In Patients #11 and #12, whose CDR staged 0 and ages were younger, had neuropsychiatric symptoms and hypoperfusion within temporal regions on Tc-99 m-ECD SPECT. Among cases whose CDR staged 0.5 or greater, hypoperfusions of dorsolateral prefrontal regions turned to be prominent as dysexcutive symptoms and attention deficits prevailed (Fig. 2).

### Neuropsychological tests

**Fig. 3:**
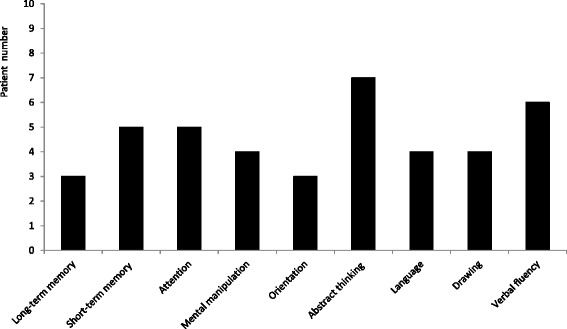
Characterization of cognitive profiles of patients with low cobalamin status

Ten patients who received complete neuropsychological tests (Patient #1 ~ #10) were further analyzed. Abstract thinking (70 %) was the cognitive domains most commonly affected, followed by verbal fluency (60 %), short-term memory (50 %), and attention (50 %) (Fig. 3). In NPI evaluations, there were three patients identified as having clinically remarkable anxiety and sleep problems (30 %). Only one patient had clinically remarkable depression (10 %). Six patients were re-examined neuropsychological tests 3 months later after Cbl replacement therapy, when normalization of serum Cbl level was achieved. Three of them had improvement (Patient #3: 24 to 28; Patient #7: 28 to 30; Patient #8: 27 to 29) but the other three (Patient #1: 12 to 11; Patient #6: 21 to 18; Patient #10: 27 to 23) had decline of MMSE total score.

### Tc-99 m-ECD SPECT follow-up studies

**Fig. 4:**
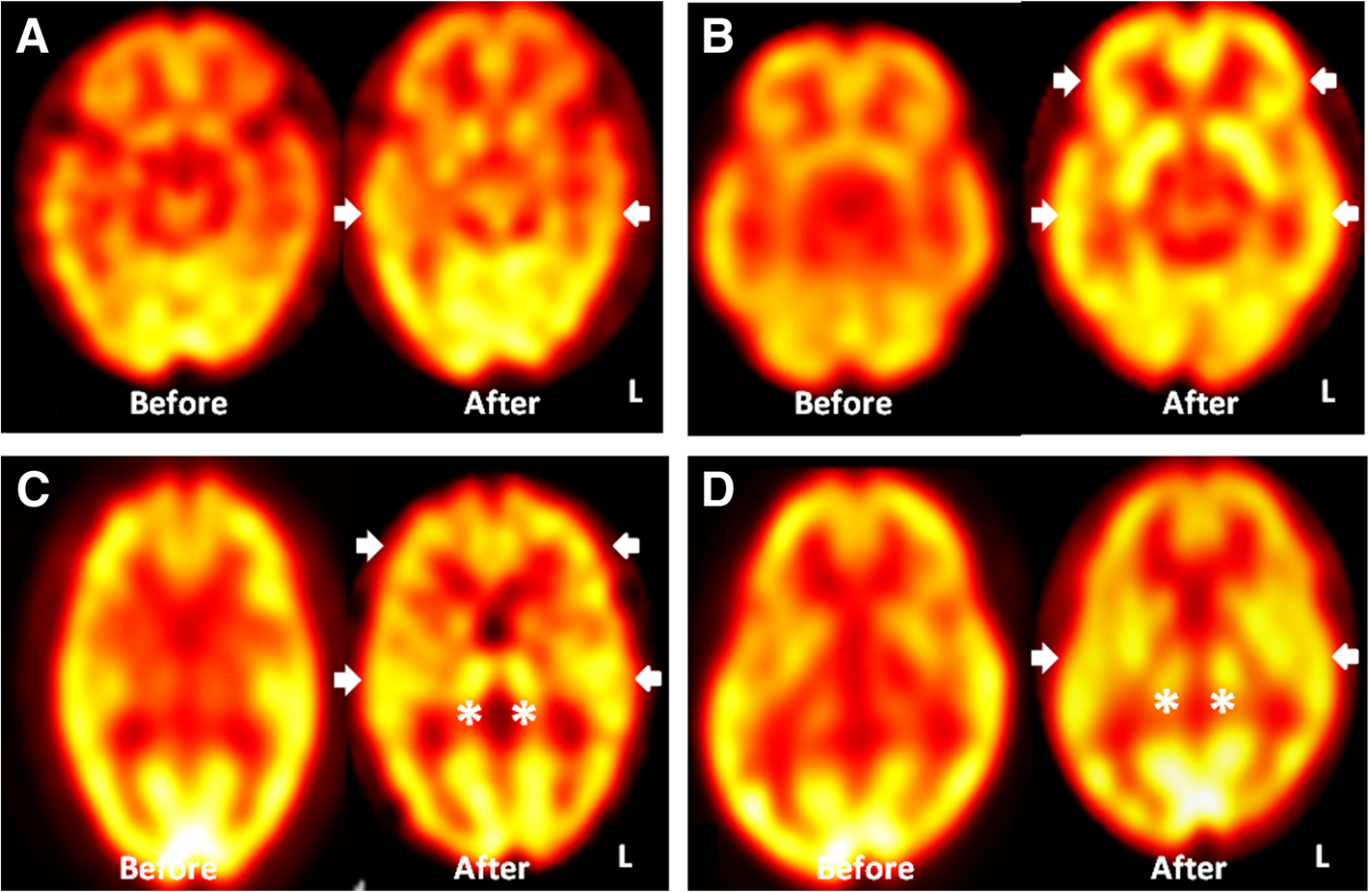
Single-photon emission computed tomography (SPECT) signal changes in relation to cobalamin replacement therapy. (**a**) A demented patient with no improvement in cognition after therapy [Mini-Mental State Examination (MMSE) = 12 to 11] had minimal signal regain only within temporal regions. (arrow heads; Patient #1) (**b**) A mildly cognitive impaired patient with improvement in cognition after therapy (MMSE = 24 to 28) had avid SPECT signal recovery within bilateral fronto-temporal regions.(arrow heads; Patient #3) (**c**) A patient with improvement of initial dysexecutive complaints and anxiety after therapy (MMSE = 28 to 30) had improving signals within bilateral thalamus (asterisk) and fronto-temporal regions.(arrow heads; Patient #7) (**d**) A mildly cognitive impaired patient with improvement in cognition after therapy (MMSE = 27 to 29) had improving perfusion within bilateral thalamus (asterisk) and temporal regions from the follow-up SPECT. (arrow heads; Patient #8)

Tc-99 m-ECD SPECT follow-up studies were available in four patients. They were proceeded three to five months after Cbl replacement therapy (Patient #1: 5 months; Patient #3: 5 months; Patient #7: 5 months; Patient #8: 3 months). In visual assessment, signals reversal was identified within most regions that were originally impaired, the extent and degree of signal reversal correlated with individual therapeutic responses (Fig. 4).

In quantitative assessment of cerebral cortices (Table 3), Patient #3, #5, #7 had global post-treatment increase over baseline value compared with Patient #1, who had increased value limited within right fronto-temporal and bilateral parietal regions. Patient #3, #5, #7 also had more intense post-treatment increase within fronto-temporal regions than Patient #1. In the assessment of deep nuclei, although all patients had signal increase, only Patient #7 and #8 had signal normalization.

**Table 3:** Regions-to-cerebellar ratio changes in relation to cobalamin replacement therapy

	Frontal regions						Temporal regions						Parietal regions						Thalamus						Basal ganglia					
	Rt			Lt			Rt			Lt			Rt			Lt			Rt			Lt			Rt			Lt		
Patient#																														
1 before	.81	.77	.78	.82	.80	.76	.80	.78	.79	.75	.75	.71	.79	.78	.79	.79	.79	.80	.76	.75	.79	.72	.73	.68	.82	.82	.81	.74	.74	.74
	**.79**			**.79**			**.79**			**.74**			**.79**			**.79**			**.77**			**.71**			**.82**			**.74**		
1 after	.79	.81	.81	.76	.76	.80	.89	.87	.88	.77	.77	.75	1.04	1.05	1.05	.95	.93	.91	.79	.78	.78	.72	.72	.72	.85	.84	.85	.74	.74	.74
	**.80**			**.77**			**.88**			**.76**			**1.05**			**.93**			**.78**			**.72**			**.85**			**.74**		
3 before	.76	.80	.81	.80	.80	.76	.75	.75	.76	.76	.78	.81	.99	1.01	.99	.95	.95	.99	.73	.72	.73	.68	.68	.68	.96	.95	.96	.92	.93	.93
	**.79**			**.79**			**.75**			**.78**			**1.00**			**.96**			**.73**			**.68**			**.96**			**.93**		
3 after	.92	.92	.90	.87	.87	.87	.92	.92	.93	.90	.86	.86	1.00	1.01	1.01	.96	.97	.98	.73	.73	.72	.74	.75	.75	.94	.94	.94	.93	.93	.92
	**.91**			**.87**			**.92**			**.87**			**1.01**			**.97**			**.73**			**.75**			**.94**			**.93**		
7 before	.78	.80	.76	.79	.77	.77	.75	.75	.74	.75	.75	.73	.85	.87	.85	.84	.81	.83	.67	.69	.69	.69	.69	.70	.91	.92	.94	.93	.95	.93
	**.78**			**.78**			**.75**			**.74**			**.86**			**.83**			**.68**			**.69**			**.92**			**.94**		
7 after	.88	.88	.82	.88	.86	.87	.81	.81	.81	.87	.84	.84	.85	.87	.88	.86	.87	.86	.80	.81	.80	.83	.82	.82	.84	.85	.86	.88	.88	.87
	**.86**			**.86**			**.81**			**.85**			**.87**			**.86**			**.80**			**.82**			**.85**			**.88**		
8 before	.84	.88	.87	.90	.92	.88	.75	.70	.70	.79	.79	.79	.90	.89	.89	.92	.92	.93	.71	.69	.72	.79	.82	.75	.91	.90	.87	.96	.95	.96
	**.86**			**.90**			**.72**			**.79**			**.89**			**.92**			**.71**			**.79**			**.89**			**.96**		
8 after	.93	.92	.94	.96	.96	.96	1.00	1.00	1.00	1.03	1.04	1.03	.95	.94	.95	1.02	1.01	.98	.86	.87	.86	.87	.87	.88	.90	.91	.91	.97	.97	.97
	**.92**			**.96**			**1.00**			**1.03**			**.95**			**1.00**			**.86**			**.87**			**.91**			**.97**		

Abbreviations: *Rt* right, *Lt* left; data were presented as ratio count from three consecutive slices (serif) followed by their average value (boldface)

## Discussion

The aim of this work is to elucidate TC-99 m-ECD SPECT findings in conjunction with clinical presentations and symptom severity among patients with Cbl deficiency. The selective involvement at bilateral fronto-temporal regions seems to be the most consistent TC-99 m-ECD SPECT findings in Cbl-deficient patients. Prefrontal lesions commonly refer to executive dysfunction and vice versa [16]. Our cohort associates dysexecutive syndrome and attention deficits with hypofrontality of TC-99 m-ECD SPECT. It’s the case that our patients with low serum Cbl status frequently encountered difficulty in planning personal agenda, managing situations of daily life, and tolerating changes of routine. There is a wealth of evidences suggesting roles of prefrontal regions in abstract thinking, verbal fluency, executive dysfunction, and attention maintenance [17, 18]. The attention recruitment, highly allied with execution network, implement newly-generated schema and later assess their accuracy whenever an individual encounter a novel situation [19]. Moreover, it is hypothesized that the prefrontal cortex serves a specific function in cognitive control, in which patterns of activity that represent goals were actively maintained by shifting attention of all sensory modalities [20]. While prefrontal areas are pivotal for executive function and attention control, several other regions, including basal ganglia [18], thalamus, and cingulate gyrus are frequently recruited to accomplish implementation of such function [21]. The dorsolateral prefrontal circuits, as one of the major connections originating from the basal ganglia, govern implicit learning, sequencing, and allocation and filtering of attention [22]. These operations would be allocated in enhancing the efficiency of higher order processors such as execution, motor planning, and mental manipulation. Deactivation of basal ganglia would impair activity of frontal regions through dorsolateral prefrontal circuits, leading dysexecutive syndrome among Cbl-deficient patients.

Our TC-99 m-ECD SPECT also addresses the clinical importance of hypoperfusion within temporal lobes. While lateral and ventral portions of temporal lobe govern sensory input processing, medial portion of temporal lobe has been linked to encoding process of memory acquisition [23]. It is therefore reasonable to expect short-term memory deficits during the neuropsychological tests. Aside from the roles on cognition, there are prolific evidences of SPECT associating temporal lobe with various psychiatric symptoms [24]. The limbic circuits of basal ganglia, constituted by reciprocal projections between ventral part of striatum and temporal lobe, have also been approved of great impact on psychiatric disorders [22]. Two young patients in our studies (Patient #11 and #12), whose ages are far below commonly-known neurodegenerative diseases, address the negative impact of Cbl deficiency on cerebral metabolism. They often prone to worry about things that are not general cause for concern. From our cohort of patients with low Cbl status, anxiety and sleep problems are the major psychiatric features, and symptoms of anxiety and irritability might predate cognitive decline. By incorporating hypofunction of basal ganglia and fronto-temporal regions as the evidences of neurobiological abnormalities, distortion of cognition would further predispose anxiety and irritability, making patients with Cbl deficiency more vulnerable to react with changes of environmental stimuli.

We are convinced that TC-99 m-ECD SPECT evaluations not only provide neurobiological measures to undergird clinical phenomenology but serve as a supplementary tool for the workup of patients with cognitive decline. Frontal regions deactivation might serve as a surrogate marker for differentiating Cbl deficiency from Alzheimer’s disease, in which radiouptake in precuneus, posterior cingulate gyrus, parietal and medial temporal lobes are preferentially impaired [24, 25]. Moreover, characterizing SPECT patterns would be of paramount clinical value before clinically-overt dementia occurs. Our study describes cognitive decline in a wide spectrum of severity, although the majority of our patients are categorized as MCI given with objective cognitive deficits but substantially preserved daily functional autonomy. Current researches frequently dichotomize MCI into amnestic and non-amnestic subtypes by neuropsychological tests. In contrast to the latter, amnestic subtype of MCI has greater possibility to share Alzheimer’s disease pathology as it preludes demented state in a closer temporal relationship [26]. The analysis of neuropsychological tests supports that non-amnestic subtype of MCI constituted as the majority of our cohort, whose TC-99 m-ECD SPECT features a pattern of symmetric hypoperfusion within frontotemporal regions. The widespread cerebral blood flow decrement not only stresses its relevance with metabolic derangement but also provides an interesting contrast to recent SPECT reports of MCI, where asymmetric hypoperfusion over frontal regions and hippocampus occurs in association with non-amnestic and amnestic subtypes, respectively [27]. Hypofrontality in our TC-99 m-ECD SPECT observations also provides an interesting parallel to another cohort study, in which predominant post-central cerebral blood flow decrement with better preserved central and prefrontal flow value is described among Cbl-deficient patients with heterogenous dementia subtypes and superimposed delirium [28]. As all of our patients with cognitive complaints are free of delirium and/or of psychotropic medications before TC-99 m-ECD SPECT evaluations, the linkage between functional neuroimaging and the cognitive deficits relevant to Cbl deficiency would be more straightforward. Still, doubt on co-existing neurodegenerative disease in our cohort might exist. We regard Cbl deficiency as the main causes of cognitive impairment in the current study based onto their structural images are nearly normal. While hypoperfusion of TC-99 m-ECD SPECT is pronounced, the volume of brain parenchyma remains fully preserved. Moreover, the signal reversal of follow-up Tc-99 m-ECD SPECT after Cbl replacement therapy supports the linkage between low Cbl status and cerebral metabolic changes. The extent and degree of signal reversal of TC-99 m-ECD SPECT, in line with cognitive changes, serves as useful internal control to validate our hypothesis.

There are several lines of mechanisms explaining symptoms attributed to Cbl deficiency. First is the derangement in the monoamine neurotransmitter, as both Cbl and folic acid are prerequisites for its production [4]. Second is impaired DNA synthesis by reducing availability of tetrahydrofolate, the essential substance maintaining cellular integrity [29]. Third is damages of vessels and myelin secondary to acumination of homocysteine and methylmalonic acid [30]. Given with nearly-normal conventional MRI images in this study, we therefore assume that neurotransmitter derangement and damage of neuronal integrity contribute to the hypometabolism of fronto-temporal regions on TC-99 m-ECD SPECT, as regional cerebral blood flow has been regarded to reflect synaptic activities [31]. Our hypothesis is also in line with previous animal experiments, issuing Cbl deficiency would impair cerebral glucose metabolism in cortical as well as subcortical areas of the brain [32].

It’s also worth to point out that the metabolic derangement among Cbl-deficient patients predates obvious changes of structural neuroimaging, even among young patients. In our cohort, there were two patients, aged 29 and 41, presented psychiatric symptoms and relevant hypoperfusion restricted within temporal regions and deep nuclei. These two patients, whose ages are much younger than those of patients with neurodegenerative disease, address evidences supporting negative impact of Cbl deficiency toward central nervous system. Some may speculate whether TC-99 m-ECD SPECT findings are the epiphenomenon related to endogenous mood disorders rather than the results relevant to Cbl deficiency. Nonetheless, their low Cbl level raises the plausible explanation to their unusual presentations, which is distinct from typical mood disorders. Moreover, half of our patients with follow-up neuropsychiatric tests show remarkable improvement after 3 months of Cbl supplement therapy. The robust findings mirror the causality between cognitive impairment and Cbl deficiency. Although poor responders to Cbl supplement exist, such findings don’t preclude the negative impact of low Cbl state, as normalization of serum Cbl level doesn’t guarantee complete restoration of cellular reserve and utilization process [1].

There are several limitations in our study. First, the study was limited in its cross-sectional study design as well as small sample size. Generalization of the results warrants future prospective studies of greater case number. Comparison of SPECT findings between groups with similar cognitive achievement but different Cbl level would also of high research value to document linkage between Cbl level and SPECT presentations. Second, as excluding co-existing neurodegenerative disease has always been a great challenge in the studies related to Cbl deficiency, we had integrated brain MRI into diagnostic consideration. We regard the possibility of co-existing Alzheimer’s disease in our cohort is limited, as our patients have no medial temporal lobe atrophy, the pathological change typically observed in Alzheimer’s disease or amnestic type of MCI. The signal reversal after Cbl replacement also served as evidences to corroborate that the underlying pathogenesis in our cohort is related to Cbl deficiency. Third, the study of other serology markers related to Cbl was not comprehensive in our study. Due to the fact that serum methylmalonic acid and homocysteine elevates earlier than the serum Cbl decrement [33] and holotranscobalamin, the biologically active Cbl, correlates with Cbl status in a more sensitive manner [34, 35], application of these serum markers in the future studies may provide further information fundamental for changes of cerebral metabolism. Last, although our TC-99 m-ECD SPECT evaluations provided convincing evidences representing functional derangement within selected regions, future studies, preferably analyzing segregated anatomical regions, may be more straightforward underpinning cerebral metabolic changes attributed to Cbl deficiency.

## Conclusion

The retrospective review of our TC-99 m-ECD SPECT observations provides pivotal information of neurobiological changes within basal ganglia and fronto-temporal regions in conjunction with disease severity among patients with Cbl deficiency. Hypoperfusion within basal ganglia in addition to temporal regions may be seen in the earlier state of Cbl deficiency, when cognition remains intact but psychiatric symptoms predominate. Hypoperfusion within basal ganglia in association with frontal regions appears when cognitive problems, mostly dysexecutive syndrome, are manifested. Symmetric hypofrontality in association with dysexcutive syndrome serves as a distinguishing feature of non-amnestic MCI attributed to Cbl deficiency, in contrast to amnestic MCI or Alzheimer’s disease. Concordant with TC-99 m-ECD SPECT findings, the psychiatric symptoms and dysexcutive syndrome undergird impaired limbic and dorsolateral prefrontal circuits originated from basal ganglia respectively. As prompt supplement reverses cognitive deficits among certain portion of patients with Cbl deficiency, judicious evaluation of Cbl status should be highlighted among patients with non-amnestic MCI, especially in the context of poor nutrition intake and/or malabsorption.

## Abbreviations

CASI: cognitive abilities screening instrument
Cbl: cobalamin
CDR: clinical dementia Rating
MCI: mild cognitive impairment
MMSE: mini-mental state examination
MRI: magnetic resonance imaging
NPI: neuropsychiatric inventory
Tc-99 m-ECD SPECT: Tc-99 m ethyl cysteinate dimer single-photon emission computed tomography
